# Energetics as a driver of human morphological thermal adaptation; evidence from female ultra-endurance athletes

**DOI:** 10.1017/ehs.2021.17

**Published:** 2021-03-29

**Authors:** Daniel P. Longman, Alison Murray, Rebecca Roberts, Saskia Oakley, Jonathan C. K. Wells, Jay T. Stock

**Affiliations:** 1 School of Sport, Health and Exercise Sciences, Loughborough University, Loughborough LE11 3TU, UK; 2 Department of Anthropology, University of Victoria, British Columbia, Canada; 3 Department of Archaeology, University of Cambridge, Cambridge CB2 3QG, UK; 4 Childhood Nutrition Research Centre, Population, Policy and Practice Research and Teaching Programme, UCL Great Ormond Street Institute of Child Health, London WC1N 1EH, UK; 5 Department of Anthropology, Western University, Ontario, Canada; 6 Department of Archaeology, Max Planck Institute for the Science of Human History, Kahlaische Strasse 10, D-07745 Jena, Germany

**Keywords:** Phenotype, adaptation, Bergmann's Rule, Allen's Rule, ecogeographical patterning, morphology

## Abstract

Functional benefits of the morphologies described by Bergmann's and Allen's rules in human males have recently been reported. However, the functional implications of ecogeographical patterning in females remain poorly understood. Here, we report the findings of preliminary work analysing the association between body shape and performance in female ultramarathon runners (*n =* 36) competing in hot and cold environments. The body shapes differed between finishers of hot and cold races, and also between hot race finishers and non-finishers. Variability in race performance across different settings supports the notion that human phenotype is adapted to different thermal environments as ecogeographical patterns have reported previously. This report provides support for the recent hypothesis that the heightened thermal strain associated with prolonged physical activity in hot/cold environments may have driven the emergence of thermally adaptive phenotypes in our evolutionary past. These results also tentatively suggest that the relationship between morphology and performance may be stronger in female vs. male athletes. This potential sex difference is discussed with reference to the evolved unique energetic context of human female reproduction. Further work, with a larger sample size, is required to investigate the observed potential sex differences in the strength of the relationship between phenotype and performance.

## Graphical abstract

**Figure:**
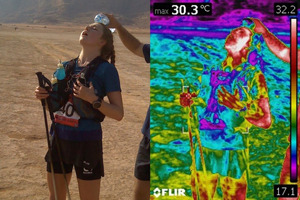

## Introduction

The ability to regulate core body temperature is a challenge that all endotherms must confront. Ecologists have long drawn insight from Bergmann's and Allen's ‘ecological rules’, which predict patterns of intra- and inter-species phenotypic variation in relation to the temperature of their environment (Allen, 1887; Bergmann, 1847). In cold climates, endotherms are proposed to be larger (Bergmann's Rule) and to exhibit shorter limbs and body appendages (Allen's Rule) compared with those found in hot climates. Both rules are based on fundamental thermodynamic principles whereby heat is produced through cellular activity in proportion to body mass, and heat is lost by radiation, convection and evaporation in proportion to surface area. Surface area increases as a square function of the linear dimension while mass increases as a cubic function, so increasing body size leads to a larger mass-to-surface area ratio. Large body sizes and relatively shorter limbs thereby provide an increased heat-producing cellular mass relative to heat-losing surface area, which is thermally advantageous in cold conditions. The inverse morphology is beneficial in hot conditions, where a reduced mass-to-surface area ratio is favoured.

Phenotypic variation consistent with Bergmann's and Allen's Rules has been observed in both extinct and extant hominin species (Crognier, 1981; Foster & Collard, 2013; Hiernaux, 1968; Hiernaux & Fromont, 1976; Holliday, 1997; Holliday & Trinkaus, 1991; Ruff, 1994; Stinson, 1990; Tilkens et al., 2007; Trinkaus, 1981). Mean annual temperature has been shown to vary negatively with body mass (Roberts, 1953) and positively with leg length in humans (Roberts, 1973, 1978). In addition to absolute size, body composition also varies with temperature, with greater abundance of heat-producing lean mass and adiposity being associated with cold conditions in both sexes (Wells, 2012). However, variability in size and shape also has implications for other functions, as we now discuss.

### The interaction between physical activity, thermoregulation and morphology

Physical activity, and endurance running in particular, may have contributed significantly to human evolution. As described by the endurance running hypothesis, a suite of human traits can be considered as adaptations enhancing long-distance running ability (Carrier, 1984), which in turn aided scavenging and hunting (Bramble & Lieberman, 2004; Lieberman et al., 2006).

While most research considering the applicability of Bergmann's and Allen's Rules to humans has been directed to external climatic sources of thermal stress, the influence of internal heat production through physical activity also merits consideration. Energetic conversion from chemical to kinetic forms during muscular contraction is inefficient, and leads to substantial amounts of heat being produced when an individual is physically active (Convertino et al., 1996; Hawley et al., 2014). This is particularly true for males, who tend to have a higher muscle mass (Welle et al., 2008) and generate more heat than females when exercising at a defined intensity (Gagnon et al., 2008). This has significant implications for thermoregulation during physical activity. In cold conditions, heat generated during periods of activity contributes towards the maintenance of core body temperature. This has the effect of reducing the potential metabolic cost of thermoregulation. Conversely, in hot conditions, heat generated by contracting muscles enhances the thermoregulatory burden and risk of hyperthermia (Ocobock, 2016a; Raynaud et al. 1976; Rivera-Brown et al., 2006).

The thermoregulatory burden of physical activity in extreme environments was evident in our recent investigation, reporting the first evidence of functional benefits conferred by thermally adapted morphologies (Longman et al., 2019). Male athletes competing in ultramarathon competitions who exhibited a greater degree of correspondence between phenotype and race conditions, according to the predictions of ecogeographic rules, were more likely to successfully complete a race in thermally challenging environments than those with morphologies less well suited to the conditions (Longman et al., 2019). We suggested that morphologies consistent with Bergmann's and Allen's Rules may reduce the metabolic burden of active thermoregulation, thereby allowing a greater energetic allocation to running, enhancing athletic performance in any given environment. This may be achieved in cold environments by reducing the sizeable metabolic costs imposed by active thermoregulation (Hill et al., 2013; Nagy, 2005; Tilkens et al., 2007), and in hot conditions by facilitating greater total energy expenditure by increasing capacity to dissipate heat and avoid hyperthermia (Speakman, 2010). However, the functional implications of ecogeographical patterning in females remain poorly understood.

### A sex difference in thermoregulatory stress during physical activity?

In contrast to many animal species, humans exhibit significant sexual dimorphism not only in body size but also in body composition, with males tending to have greater lean mass and lower fat mass relative to body weight than females (Wells, 2007). This has potential implications for the heat stress conferred by physical activity in hot/cold environments, as both tissues make important contributions to thermoregulation (Garby et al., 1988).

Skeletal muscle is a key thermogenic tissue, capable of generating significant amounts of heat when active (González-Alonso, 2012; Pant et al., 2016; Rowland et al., 2015). In cold conditions skeletal muscle promotes thermal balance when active through heat production, providing protection from heat loss and cold injury (Payne et al., 2018). When inactive and vasoconstricted, muscle provides a substantial layer of insulation between the core and skin surface (60–80% in lean subjects). The insulative properties of muscle decrease markedly upon activation, falling to 10–20% of maximal insulation value (Rennie, 1988).

Adipose tissue stores energy for maintaining core body temperature in cold conditions (Cannon & Nedergaard, 2004; Nedergaard et al., 2007), particularly thermogenic abdominal fat (Beall & Goldstein, 1992). The general pattern for increased muscularity and adiposity of populations exposed to cold stress (Craig et al., 2001; Gnaiger et al., 2015; Houghton, 1990; Jones & White, 1994; Piers et al., 2003) highlights the thermal properties of skeletal muscle and adipose tissue (McArdle et al., 1984). The insulating role of fat has not been definitively demonstrated and remains questionable. For example, historical data from the most northerly population in the world (the Greenland Thule) showed very low skinfold thicknesses (Gilberg, Jørgensen, & Laughlin, 1975), and a study of female Korean cold-water divers revealed reduced subcutaneous fat in relation to other Korean women (Rennie et al., 1962). However, experimental evidence suggests that fat does have insulating properties, and that the degree of insulation conferred by subcutaneous adiposity varies negatively with blood flow to the skin (Burse, 1979; Havenith, 1997; Havenith et al., 1998). Given the heat-producing and potentially insulating qualities of muscle and fat tissue, one might expect individuals with low lean and fat mass to experience lower thermal strain in hot conditions.

Such sexually dimorphic morphological features are believed to have arisen from the unique energetic context of human female reproduction. Human females store energy in advance to allow the funding of reproduction irrespective of dietary energy availability (Jönsson, 1997). Prior to reproduction, fat is stored predominantly in the gluteo-femoral region, with further fat stores accumulated during pregnancy. Both pregnancy and lactation require the mother to provide a steady source of nutrition for her offspring to meet a metabolic demand heightened by the evolution of the enlarged brain (Holliday, 1971). While fetal growth is energetically funded primarily from maternal glucose dynamics, fat accumulation during pregnancy stores energy to support infant nutrition through lactation (Wells, 2018). This, in turn, favours higher levels of body fat in females relative to males.

### The current study

The current study recruited female participants in multiday ultramarathon competitions in hot and cold climates to investigate the relationship between body shape and endurance running performance in thermally challenging environments. This study was part of a wider body of research recruiting athletes as a model to investigate evolutionary theory (Longman et al., 2015, 2017a, 2018). This approach was adopted by the ADaPt Project, which utilised ultra-endurance athletic events to investigate life history trade-offs (Longman et al., 2017b).

Multiday ultra-endurance contests impose high locomotor energetic demands. Even in the absence of physical activity, thermoregulation is a metabolically expensive process (Hill et al., 2013). Experimental work with lizards demonstrated a trade-off between thermoregulation and growth rate (Brewster et al., 2013). Although not an efficient long-term physiological solution to cold exposure, human shivering causes the metabolic rate to increase by a factor of 5–6 compared with resting levels (Glickman et al., 1967; Iampietro et al., 1960; Keatinge et al., 1986; Parsons, 2003). In hot conditions, basal metabolic has been shown to increase when the environment is humid, but decrease when it is dry and sweating is effective (Chinevere et al., 2008; Hori, 1995; Osiba, 1957; Shapiro et al., 1980).

Here, it is expected that the performance of female endurance athletes competing in hot and cold environments will correspond with thermally ‘adaptive’ body types, serving to minimise thermal strain by preserving and dissipating heat in cold and hot environments respectively. We recently reported similar trends in male runners (Longman et al., 2019). Irrespective of sex, all athletes had to overcome the thermoregulatory challenges associated with completing the same racecourse in the same environment. Female athletes exhibiting a greater degree of morphological thermal adaptation are expected to have a reduced thermoregulatory metabolic burden (Tilkens et al., 2007), allowing for greater energetic allocation towards running and enhanced performance.

It is hypothesised that:

1. Female athletes who successfully complete ultramarathons in hot conditions will exhibit heat-adapted morphologies relative to those who successfully complete ultramarathons in cold conditions.
2. Female athletes who successfully complete a hot-condition ultramarathon will exhibit heat-adapted morphologies relative to athletes who fail to complete the same race.

In addition, we aimed to collect preliminary evidence to begin to determine whether there is a sex difference in the relationship between performance and morphology (using comparative data previously reported in Longman et al., 2019).

## Methods

### Experimental design

Female (*n* = 36) and male (*n* = 88) athletes were tested at ultramarathon races across hot (the 2016 Jungle Ultra, Peru, and the 2016 and 2017 Al Andalus Ultimate Trail, Spain) and cold (2016 Rovaniemi150, Finland, and the 2016 Everest Trail Race, Nepal) climatic conditions. All but one of the athletes were of European ancestry and travelled to the country hosting the event. Except for the Finland event, which was continuous, the remaining three events employed a similar format of five or six consecutive daily stages with an overnight rest in between. An overview of the races can be seen in Table 1.

**Table 1.** Overview of the four ultramarathons (taken from Longman et al., 2019)

Race	Rovaniemi150*, Finland	Jungle Ultra, Peru	Al Andalus Ultimate Trail, Spain	Everest Trail Race, Nepal
Location	Rovaniemi, Finland	Manu National Park, Peru	Granada Province, Spain	Solukhumbu District, Nepal
Latitude	66°N	12°S	37°N	27°N
Race dates	Late February	Early June	Mid-July	Mid-November
Race format	Continuous	5 stages	5 stages	6 stages
Distance	66/150 km	250 km	230 km	153 km
Elevation change	1,356 m ascent, 1,350 m descent	1,800 m ascent, 3,200 m descent	7,100 m ascent, 7,100 m descent	13,500 m ascent, 13,000 m descent
Elevation: Start/finish	200 m/200 m	2900 m/1500 m	450 m/450 m	2370 m/2860 m
Climate	Cold (2 to −11°C)	Hot and humid (30°C, 77%)	Hot and dry (35–45°C)	Cool days (5–15°C), cold nights (−10°C)

*Athletes were able to complete the Rovaniemi150 on foot, on skis or by bike. Only runners were included in this analysis.

Tables 2 and 3 provide a description of the sample cohort by competition and condition, as well as race completion times by condition.

**Table 2.** Descriptive characteristics for all participating athletes, split by competition (male data replicated from Longman et al., 2019)

Metric	Sex	Finland Mean (SD), *n* = 16 male, 2 female	Peru Mean (SD) *n* = 15 male, 7 female	Spain Mean (SD) *n* = 38 male, 20 female	Nepal Mean (SD) *n* = 19 male, 7 female
Age (years)	Male	38.1 (9.6)	41.5 (7.9)	49.2 (9.7)	46.3 (11.7)
	Female	53.5 (6.4)	35.7 (10.3)	47.8 (9.1)	40.1 (5.01)
Height (cm)***	Male	180.8 (5.1)	177.9 (4.5)	180.3 (6.9)	176.1 (6.0)
	Female	163.5 (12.7)	162.6 (5.9)	166.1 (5.2)	165.5 (6.4)
Weight (kg)***	Male	80.8 (5.5)	80.8 (7.4)	78.6 (8.8)	78.1 (10.0)
	Female	65.4 (13.8)	60.7 (4.1)	61.7 (7.3)	65.7 (8.5)
BMI (kg/m^2^)***	Male	24.7 (1.5)	25.5 (1.9)	24.1 (2.1)	25.2 (2.8)
	Female	24.3 (1.4)	23.0 (.8)	22.3 (2.3)	23.9 (1.9)
Ponderal index	Male	13.7 (1.0)	14.3 (1.1)	13.4 (1.3)	14.3 (1.7)
	Female	14.9 (.3)	14.1 (.8)	13.5 (1.5)	14.4 (1.0)
Waist circumference (cm)***	Male	80.9 (7.2)	85.4 (6.3)	85.6 (5.8)	85.7 (8.3)
	Female	74.3 (3.9)	71.6 (2.6)	74.3 (5.5)	74.9 (8.7)
Hip circumference (cm)*	Male	98.4 (5.1)	101.3 (3.4)	100.4 (5.3)	100.8 (5.4)
	Female	96.3 (15.2)	98.1 (3.8)	97.2 (5.7)	101.4 (4.5)
Leg length (cm***	Male	86.1 (4.1)	88.6 (3.6)	90.2 (4.3)	84.9 (4.5)
	Female	77.0 (6.4)	78.5 (3.9)	81.7 (3.6)	79.3 (3.3)
Relative leg	Male	47.6 (1.2)	49.8 (1.2)	50.0 (1.4)	48.2 (1.1)
Length (%)	Female	47.1 (.2)	48.3 (1.0)	49.1 (1.2)	47.9 (.8)
Race completion rate	Male	62%	89%	50%	72%
	Female	80%	100%	65%	80%

Asterisks indicate a significant sex difference (* *p* < 0.05, ** *p* < 0.01, *** *p* < 0.001) between the male and female athlete cohort as a whole.

**Table 3.** Mean and standard deviations of the race time of finishers in hot and cold condition races (male data replicated from Longman et al., 2019)

Sex	Hot conditions, mean (SD), (h:min:s)	Cold conditions, mean (SD) (s)	*t*	*p*
Female	35:45:27 (5:34:24)	36:55:40 (18:09:15)	−0.256	0.800
Male	34:23:29 (5:20:08)	34:16:37 (22:52:01)	0.028	0.978

### Anthropometrics

Previously studies have used a variety of measures to assess Bergmann's Rule, including body mass, height, body mass index (BMI) and ponderal index, as well as hip and waist circumferences (Foster & Collard, 2013; Ruff, 1994). There is currently no consensus regarding which specific variables should be used to consider the relationship between morphology and environmental temperature (Foster & Collard, 2013). In this study, each of these measures was taken to examine the relationship between body size and race condition (circumferences were preferred to bi-iliac breadth owing to perceived superior accuracy).

Stature was measured to the nearest 0.1 cm using a Leicester stadiometer. Body mass (kg) was measured to the nearest 0.1 kg (Seca, Hamburg, Germany). Waist and hip circumferences (cm) and sitting height (cm) were measured according to the standards in the International Standards for Anthropometric Assessment (2001). An index of leg length was obtained by subtracting sitting height from stature, and relative leg length was obtained by dividing leg length by stature. Body mass index was calculated as body mass (kg) divided by the square of stature (m). Ponderal index was calculated as body mass (kg) divided by the cube of stature (m).

### Defining athletes’ performance

Athletes’ performance within each race was defined using the method reported in Longman et al. (2019). Athletes were categorised as finisher or non-finisher. Finishers were those who completed the full race distance within the time limits set by race organisers, whereas non-finishers did not.

The process of heat acclimatisation can provide short-term biological adaptations acting to reduce the negative effects of heat stress. Improvements in factors such as cardiovascular stability, fluid-electrolyte balance, sweating and skin surface blood flow promote submaximal and maximal aerobic performance in hot conditions (see Longman et al., 2019). Analysis of questionnaire data (which detailed athletes’ pre-race acclimatisation) found only two female athletes who could be categorised as heat-acclimatised, and none who could be considered cold-acclimatised (Pandolf, 1998; Sawka et al., 2012), and there were no significant differences in the morphologies between the heat-adapted and non-heat-adapted cohorts.

### Statistical analysis

One-way analysis of covariance (ANCOVA, controlling for age) was used to identify any statistically significant differences between athletes who finished races in both conditions, and between athletes who did and did not finish races in hot conditions. To address Hypothesis 2, data were collected from two consecutive editions of the Spain ultra-endurance event in order to obtain a sample size allowing this analysis. Owing to sample size constraints, it was not possible to do the same for cold conditions. Graphs were produced by expressing differences between groups as a percentage (the group-difference in natural log-transformed values of each measure was multiplied by 100%). In order to assess differences between athletes who did and did not finish the Spain event, one-way analysis of covariance (ANCOVA, again controlling for age) was used.

Independent samples *t*-tests (one-tailed) were performed to test for sex differences in the baseline variables. The data was checked for normality and homogeneity of variance, and all data met parametric assumptions. Consistent with previous reports of an age effect on endurance running performance (Cejka et al., 2015; Hoffman & Fogard, 2011; Hunter et al., 2011; Rüst et al., 2013, 2014), race finishers were significantly younger than those who did not finish (41.1 ± 9.21 vs. 52.4 ± 6.79 years, *t*(34) = −3.512, *p* = 0.001). Consequently, age was controlled for in subsequent analyses. We also controlled for the potential confounding effect of altitude on performance (using the mean average of total ascent and descent).

In addition, multiple linear regression was performed to further analyse morphological differences between athlete groups, and a logistic regression was performed to determine the effects of morphological variables on the likelihood that an athlete finished the race. SPSS v25 was used for all analyses, with a significance benchmark of 0.05.

This project was granted ethical approval by the University of Cambridge Human Biology Ethics Committee (HBREC.2016.14). Written informed consent was obtained, and all methods were carried out in accordance with the relevant guidelines and regulations. Data will be made available via Loughborough University's online Research Repository.

## Results

Hypothesis 1: Female athletes who successfully completed ultramarathons in hot conditions will exhibit heat-adapted phenotypes (lower body mass, lower BMI, longer leg lengths) relative to those who successfully completed ultramarathons in cold conditions.

Comparison of the morphologies of all race entrants (irrespective of whether they finished their race) in hot-condition races with those in cold-condition races found that although there were no significant differences in either weight or BMI, significant differences in relative leg length (hot 48.9 ± 1.2% vs. cold 47.6 ± 0.7%, *F*(1,35) = 10.511, *p* = 0.003) suggest that there may have been a degree of self-selection to race choice, perhaps based on perceptions of differential ability in the hot and cold as well as prior experience.

**Figure 1.**
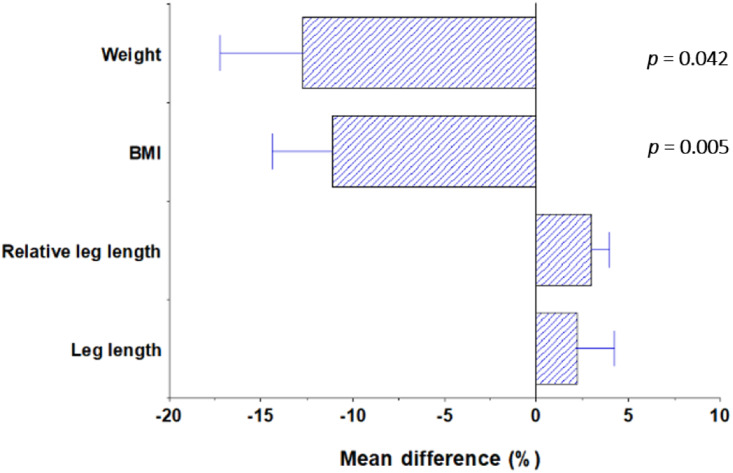
Percentage differences in anthropometric traits between race finishers in hot and cold conditions. Negative values reflect the variable being greater in cold-condition finishers than hot-condition finishers. *P*-values are provided for statistically significant differences.

The morphology of race finishers in hot vs. cold conditions was compared using one-way ANCOVA, controlling for age and total ascent/descent over the race. Female finishers of hot-condition races (*n* = 18) had lower weights and lower BMIs than those who finished a cold-condition race (*n* = 8) (weight hot 59.3 ± 6.4 kg vs. cold 67.1 ± 8.1 kg, *F*(1,22) = 4.652, *p* = 0.042; BMI hot 21.8 ± 2.0 vs. cold 24.2 ± 1.6, *F*(1,22) = 10.006, *p* = 0.005). Furthermore, female athletes who finished a hot-condition race had greater relative leg lengths than those who finished a cold-condition race (hot 49.0 ± 1.3 vs. cold 47.6 ± 0.7, *F*(1,23) = 12.321, *p* = 0.002). These results persisted following Bonferroni corrections and were consistent with both Allen's and Bergmann's Rules. These results are summarised in Figure 1.

Multiple linear regression was performed to further analyse morphological differences between athlete groups. Predictors were race type (hot and cold), finishing status (finisher and non-finisher) and the interaction between race type and finishing, while also controlling for age and total ascent/descent.

Considering BMI, a significant regression equation was found, (F(5,30) = 3.287, *p* = 0.017), with an *R*^2^ of 0.354. The interaction between race type and finishing status significantly predicted BMI, such that, taking into account overall associations with race type and finishing status, BMI was 4.2 kg/m^2^ lower in finishers of hot races compared with finishers of cold races (*t* = −2.055, *p* = 0.049). The other variables did not independently predict BMI.

The same pattern was evident for sitting height – a significant regression equation (*F*(5,30) = 2.156, *p* = 0.048), with an *R*^2^ of 0.259. The interaction between race type and finishing status significantly predicted sitting height, which was 8.6 cm greater in finishers of hot races compared with finishers of cold races (*t* = −2.670, *p* = 0.012).

Additionally, the interaction between race type and finishing status was a significant predictor of both sitting height (8.8 cm greater in finishers of hot races, *t* = −2.676, *p* = 0.012) and waist circumference (13.6 cm lower in finishers of hot races, *t* = −2.214, *p* = 0.034). The interaction between race type and finishing status did not predict other morphological variables.

Hypothesis 2: Female athletes who successfully complete a hot-condition ultramarathon will exhibit heat-adapted morphologies relative to athletes who failed to complete the same race.

Data were collected from two consecutive editions of the Spain ultra-endurance event in order to obtain a sample size allowing this analysis. The same race was used twice to test Hypothesis 2 in order to standardise the race route, and to minimise differences in weather conditions. This within-condition analysis was not performed for cold-condition events owing to the smaller available sample size. As race finishers were significantly younger than those who did not finish (44.8 ± 8.4 vs. 53.3 ± 8.1 years, *t*(18) = −2.185, *p* = 0.042), the second hypothesis was assessed using a one-way ANCOVA, controlling for age.

**Figure 2.**
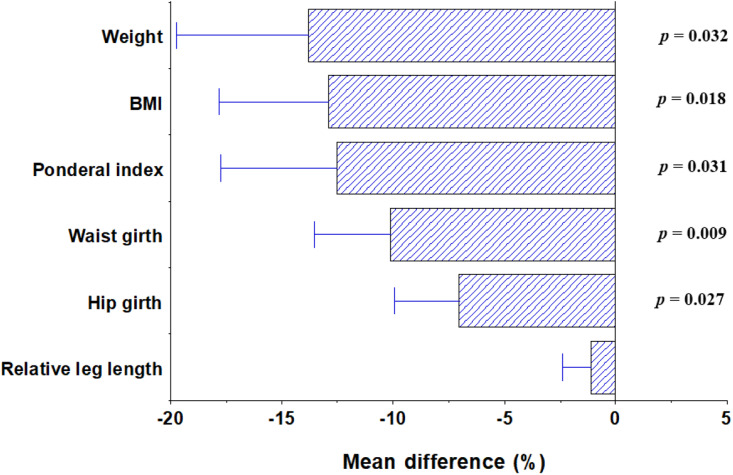
Natural log transformation of the differences between finishers and non-finishers in a hot environment. Negative values reflect the variable being greater in cold-condition finishers than hot-condition finishers. *P*-values are provided for statistically significant differences.

Female finishers were significantly lighter (59.2 ± 7.2 vs. 66.4 ± 5.0 kg, *F*(1,17) = 5.488, *p* = 0.032) and had lower BMIs (21.4 ± 2.20 vs. 24.1 ± 1.43 kg/m^2^, *F*(1,17) = 6.896, *p* = 0.018), lower ponderal indices (12.9 ± 1.47 vs. 14.5 ± 1.1, *F*(1,17) = 5.568, *p* = 0.031), smaller waists (72.3 ± 5.6 vs. 78.1 ± 2.9 cm, *F*(1,17) = 8.593, *p* = 0.009) and smaller hips (95.4 ± 6.2 vs. 100.6 ± 2.5 cm, *F*(1,17) = 5.834, *p* = 0.027) than non-finishers (see Figure 2).

A logistic regression was performed to determine the effects of morphological variables on the likelihood that an athlete finished the race. Controlling for age and when entered into the model independently of each other, weight (exp(*B*) = 0.837, *p* = 0.041), BMI (exp(*B*) = 0.487, *p* = 0.024), waist circumference (exp(*B*) = 0.693, *p* = 0.041) and hip circumference (exp(*B*) = 0.740, *p* = 0.035) were all statistically significant predictors, with higher values in each metric being associated with a lower likelihood of finishing a hot condition race. When all four measurements were added simultaneously, none of them remained significant; greater values therefore predict likelihood of finishing, but we cannot state which of the various measurements is key to this relationship.

Preliminary exploratory analysis: Is there a sex difference in the relationship between performance and morphology.

The relationship between thermally adapted body shape performance appeared, tentatively, to be more pronounced in female than male athletes (Longman et al., 2019). The associations between female morphology and performance were more pronounced, and achieved statistical significance for measures of both Bergmann's (weight, BMI, hip circumference, waist circumference and ponderal index) and Allen's Rules (relative leg length). In contrast, the relationship between male morphology and performance only achieved statistical significance in measures relating to Allen's Rule (leg length and relative leg length), and not Bergmann's Rule. These results are summarised in Table 4. However, the sample size was too small at this stage to subject the sex comparison to a formal sex-interaction test.

**Table 4.** Morphological characteristics of hot race finishers relative to (a) cold race finishers and (b) hot race non-finishers). Bold text signifies statistically significant differences between athletes

Male	Female
*(a) Hot race finishers relative to cold race finishers*	
Lighter	**Lighter**
Lower BMI	**Lower BMI**
**Longer legs**	Longer legs
**Longer relative leg length**	**Longer relative leg length**
*(b) Hot finishers vs.. hot non-finishers*	
Heavier	**Lighter**
Lower BMI	**Lower BMI**
Smaller hip circumference	**Smaller hip circumference**
Smaller waist circumference	**Smaller waist circumference**
Lower Ponderal Index	**Lower ponderal index**
**Longer relative leg length**	Longer relative leg length

The data are available online via https://www.lboro.ac.uk/departments/ssehs/staff/danny-longman.

## Discussion

The performance of female endurance athletes competing in hot and cold environments was associated with thermally beneficial body shapes. Controlling for age and racecourse elevation change, female finishers of hot-condition ultramarathons had longer legs, lower weights and lower BMIs than finishers of cold-condition events (Hypothesis 1). Furthermore, the finishers of hot-condition events had lower BMIs, hip circumferences, waist circumferences and ponderal indices, and longer legs, than athletes who failed to complete the same hot-condition events (Hypothesis 2). Energy and thermoregulation are interconnected, such that the increase in work necessary to transport a larger body around a race course results in greater heat production. This will decrease thermoregulatory strain in cold conditions, and increase it in hot conditions (Ocobock, 2016b).

### Prolonged physical exertion as a driver of morphological thermal adaptation

**Figure 3.**
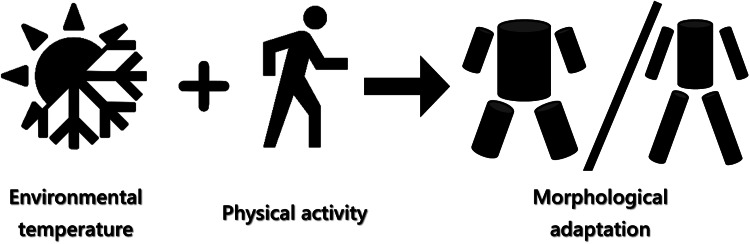
The selective pressure for the generation of thermally adapted morphologies arises from prolonged physical activity in thermally challenging environments. Taken from (Longman et al., 2019).

This study observed that female athletes with thermally adapted morphologies tended to out-perform those without such adaptations in thermally challenging environments. This provides support for the suggestion that, as exercise increases the pressures imposed by the thermal environment, the interaction between environment and endurance activity increased the strength of selection for temperature-adapted morphologies (Longman et al., 2019). Physical activity in extreme temperatures places stress on the thermoregulatory system, increasing the demand for heat loss/retention (although physical activity in cold climates can reduce the energy demands of thermogenesis; Ocobock, 2016a). This amplifies a significant selective force which could have led to the emergence of the morphologies described by Bergmann's (1847) and Allen's (1887) Rules, as opposed to adaptation to the environment allowing for subsequent physical activity (see Figure 3). The potential mechanisms underpinning morphological climatic variation are unclear, and are discussed in more detail in our recent paper (Longman et al., 2019).

### Are sex differences indicative of sex-specific evolutionary energetic trajectories?

Our results were consistent with the patterns we have previously reported in male runners (Longman et al., 2019). Irrespective of sex, all athletes had to overcome the thermoregulatory challenges associated with completing the same racecourse in the same environment. Owing to the high energetic costs of active thermoregulation (Hill et al., 2013; Nagy, 2005), greater correspondence between phenotype and race conditions according to the predictions of ecogeographic rules was hypothesised to promote athletic performance owing to the possibility for increased energy availability for locomotion.

Our preliminary work shows that female athletes appeared to show stronger associations between shape and performance. Although both male and female performance exhibited trends consistent with Bergmann's and Allen's rules, the relationship appeared to be more pronounced in female than male athletes. Despite the smaller sample size, the female subgroup revealed a greater number of statistically significant associations, spanning both Bergmann's (weight, BMI, hip circumference, waist circumference and ponderal index) and Allen's (relative leg length) Rules. In contrast, the only statistically significant relationships between male morphology and performance related to Allen's Rule (leg length and relative leg length) (Longman et al., 2019). These results suggest that climate-adaptive morphologies may promote endurance performance in female athletes to a greater degree than in male athletes. The relative thermoregulatory homeostatic restraints experienced by female athletes, which may stem from reduced muscle mass and increased adiposity, may have led to this closer relationship between morphology and performance. It is important to note, however, that the sample size did not allow for statistical analysis of this difference. Our suggestion of a sex difference is preliminary and requires further investigation.

Unlike many animal species, humans exhibit significant sexual dimorphism in body composition. In addition to being approximately 7% taller (Gustafsson & Lindenfors, 2004) and having increased bone mineral content relative to females (Maynard et al., 1998), males tend to have greater lean mass and lower relative fat mass (although similar in absolute mass) (Wells, 2007). These differences have implications for thermoregulation during physical activity in thermally challenging environments. Sex differences in thermoregulatory responses to cold conditions arise primarily from variation in anthropometry and body composition (Castellani & Young, 2016). We speculate that this may relate to the high costs of fat and lean mass during exercise. Although females tend to have more subcutaneous fat than males, lower average lean tissue mass reduces capacity for heat generation, leading to more rapid net heat loss in severely cold conditions (McArdle et al., 1984). In contrast, sex differences in the thermoregulatory response to hot conditions may be attributable to sexual dimorphism unrelated to differences in body composition. Lower female thermosensitivity in the whole-body sudomotor response when hot (Gagnon & Kenny, 2011), combined with reduced sweat gland output at high exercise intensity (Gagnon & Kenny, 2012), may result in a reduced capacity to dissipate heat by sweating.

An adaptive perspective, considering dimorphism in sex-specific evolutionary trajectories relating to energetic biology, may further explain the potential sex difference identified in this investigation. Pregnancy and lactation require the mother to provide a steady source of nutrition for her offspring (Cunnane & Crawford, 2003). During this key developmental phase, the neonate is particularly vulnerable to interruptions in energy supply (Cunnane & Crawford, 2003). While fetal growth is energetically funded primarily from maternal glucose dynamics, fat accumulation both before and during pregnancy ring-fences energy for the infant's development. This became necessary in the *Homo* lineage upon the evolution of an enlarged brain, which brought an increased metabolic demand, particularly in infancy (Kuzawa et al., 2014). By accumulating fat tissue, the mother can uncouple the baby's energy supply from ecological fluctuations, promoting her own fitness. Consequently, the increased adiposity of human females in comparison with males is indicative of the important role played by reproductive energetics in our evolutionary past (Wells, 2006). It is known that the selective pressures that favoured female adiposity in order to nourish large-brained infants may thereby have led to sexual dimorphic body composition, causing greater sensitivity to thermal stress. This may provide an explanation for the sex differences in the relationship between ultra-endurance performance and thermally adapted morphologies observed in this study.

Sexual dimorphism in body size, composition and sudomotor response suggest that males and females may differ in terms of the magnitude of homeostatic response to thermal stress when performing physical activity in hot and cold environments. In order to achieve thermal homeostasis when performing endurance activity in hot and cold environments, we predict that selective pressures acting on maternal energetics drive broader patterns of thermal adaptation within our species. As a result, ecogeographical morphological adaptation may play a more significant role in female than male athletes. While we observed differences in thermoregulation between males and females, this forms only one component of the myriad physiological variations underpinning performance.

Although significant, thermoregulation is only one biological process contributing to ultramarathon performance. Sex differences are also apparent regarding metabolism, with female physiology potentially providing a number of advantages. For example, at a given exercise intensity, female athletes may utilise proportionately more fat and less carbohydrate then male athletes (see Deaner et al., 2015). Further, female athletes are generally less susceptible to muscle glycogen depletion, and are less fatigable than males when performing sustained and intermittent isometric contractions at a similar intensity (Hunter, 2014).

It is important to note that the female sample size included within this study is relatively small, and did not allow for a statistical analysis of any sex differences that may be present. Further work, with larger sample sizes, is required to explore this difference in more detail. Although female participation in ultra-endurance athletic events has traditionally been lower than male participation (Knechtle et al., 2011), this is changing, and in the coming years it will be possible to build a larger database of female competitors. However, despite the small sample size, we observed stronger associations in females. Our findings may therefore be considered conservative, as the larger sample size in males increased the chances of obtaining significant findings.

A larger female sample size would allow for investigation of the influence of menstrual cycle phase on the relationship between endurance performance and morphology. The menstrual cycle is accompanied by rhythmic changes in core body temperature, believed to be driven by fluctuations in reproductive hormones. Both at rest and during exercise, estrogen and progesterone influence body temperature and thermoregulation. During the preovulatory follicular phase, estrogen promotes vasodilation, heat dissipation and a lower body temperature during exercise. During the mid-luteal phase, elevated progesterone levels have the opposite effect (Stephenson & Kolka, 1999). Although it has not yet been demonstrated, the effects of estrogen in reducing the thermoregulatory set point may decrease the risk of hyperthermia, while during the mid-luteal phase the progesterone-induced elevation of core body temperature enhances risk (Charkoudian & Stachenfeld, 2014).

Care was taken in selecting a series of ultramarathons which would represent as wide a range of environmental conditions as possible (Foster & Collard, 2013), whilst keeping factors such as duration and distance consistent. While we were able to control for elevation change during each race (as measured by total ascent and descent), we were unable to control for average altitude as this data was not available.

## Conclusions

To conclude, thermally advantageous body types were associated with better performance among female ultramarathon runners competing in thermally challenging environments. Phenotypic variation corresponding with predictions based upon ecogeographic rules (Allen, 1887; Bergmann, 1847) was identified in the finishers of hot-condition events in comparison with the finishers of cold-condition events, and in the finishers of hot-condition events relative to non-finishers. This provides support for the hypothesis that a heightened pressure for thermal balance stemming from prolonged physical activity in hot/cold environments drove the emergence of thermally adaptive morphologies (Longman et al., 2019).

Furthermore, a possible sex difference exists in the strength of the relationship between body shape and performance. We speculate that this may relate to sex differences in thermoregulatory pressure in such environments. Sex differences in thermoregulatory capacity may stem from differential selective forces in our evolutionary past, arising from the need for female fat accumulation to buffer infant energy supply from ecological fluctuations.
